# Ivermectin to prevent hospitalizations in patients with COVID-19 (IVERCOR-COVID19): a structured summary of a study protocol for a randomized controlled trial

**DOI:** 10.1186/s13063-020-04813-1

**Published:** 2020-11-24

**Authors:** Julio Vallejos, Rodrigo Zoni, Maria Bangher, Silvina Villamandos, Angelina Bobadilla, Fabian Plano, Claudia Campias, Evangelina Chaparro Campias, Fernando Achinelli, Hector A. Guglielmone, Jorge Ojeda, Fernanda Medina, Diego Farizano Salazar, Gerardo Andino, Natalia E. Ruiz Diaz, Pablo Kawerin, Elba Meza, Silvana Dellamea, Antonia Aquino, Victor Flores, Carolina N. Martemucci, María Mercedes Vernengo, Silvina María Martinez, Juan Emanuel Segovia, María Gabriela Aguirre

**Affiliations:** 1 Instituto de Cardiología de Corrientes “Juana F. Cabral”, Corrientes, Argentina; 2 Ministerio de Salud Pública de la Provincia de Corrientes, Corrientes, Argentina

**Keywords:** COVID-19, Randomized controlled trial, Protocol, Ivermectin, Hospitalization

## Abstract

**Objectives:**

To assess the efficacy of ivermectin in addition to standard treatment compared to standard treatment alone in reducing hospitalizations in the COVID-19 patient population.

**Trial design:**

IVERCOR-COVID19 will be a single-center, prospective, randomized, double-blind, parallel group (1:1 ratio), placebo-controlled study.

**Participants:**

Patients who meet the following criteria will be invited to participate:

Inclusion criteria: (1) Over 18 years of age who reside in the province of Corrientes at the time of diagnosis. (2) Confirmed diagnosis of COVID-19 by polymerase chain reaction (PCR) test for detection of SARS-CoV2 in the last 48 h. (3) In the case of women of childbearing age, they must be using a contraceptive method of proven efficacy and safety (barrier, hormonal, or permanent contraceptives) for at least 3 months prior to inclusion in the present study and for the entire period of time for the duration of the study and until at least 30 days after the end of this study. A woman will be considered to have no reproductive capacity if she is postmenopausal (at least 2 years without her menstrual cycles) or if she has undergone surgical sterilization (at least 1 month before the time of inviting her to participate in this study). (4) Weight at the time of inclusion greater than 48 kg. (5) That they sign the informed consent for participation in the study.

Exclusion criteria: (1) pregnant or breastfeeding women; (2) known allergy to ivermectin or some of the components of ivermectin tablets or placebo; (3) current use of home oxygen; (4) require hospitalization due to COVID-19 at the time of diagnosis or history of hospitalization for COVID-19; (5) presence of mal-absorptive syndrome; (6) presence of any other concomitant acute infectious disease; (7) known history of severe liver disease, for example liver cirrhosis; (8) need or use of antiviral drugs at the time of admission for another viral pathology other than COVID-19; (9) need or use of hydroxychloroquine or chloroquine; (10) use of ivermectin up to 7 days prior to randomization; (11) patients on dialysis or who have required it in the last 2 months or who plan to do it in the next 2 months; and (12) current participation or in the last 30 days in a research study that has included the administration of a drug (Table 1).

**Table 1:** Ivermectin/placebo dose according to patient weight

Patient weight	Ivermectin/placebo dose	Total dose (mg)
Equal to or greater than 48 kg and less than 80 kg	2 tablets of 6 mg each at the time of inclusion and 2 tablets 24 h after the first intake	24
Equal or greater than 80 kg and less than 110 kg	3 tablets of 6 mg each at the time of inclusion and 3 tablets 24 h after the first intake	36
Equal or greater than 110 kg	4 tablets of 6 mg each at the time of inclusion and 4 tablets 24 h after the first intake	48

The study will be carried out by the Ministry of Public Health of the Province of Corrientes (Argentina) in coordination with the Institute of Cardiology of Corrientes in the Province of Corrientes, Argentina.

**Intervention and comparator:**

Intervention group: patients who are randomized to ivermectin will receive the dose according to their weight (patients up to 80 kg will receive 2 tablets of 6 mg ivermectin; patients with more than 80 kg and up to 110 kg will receive 3 tablets of 6 mg of ivermectin; patients weighing more than 110 kg will receive 4 tablets of 6 mg ivermectin) the day they enter the study and the same dose 24 h after the first dose.

Control group: patients who are randomized to placebo will receive the dose according to their weight (patients up to 80 kg will receive 2 tablets of 6 mg placebo; patients with more than 80 kg and up to 110 kg will receive 3 tablets of 6 mg of placebo; patients weighing more than 110 kg will receive 4 tablets of 6 mg placebo) on the day they enter the study and the same dose 24 h after the first dose (Table 2).

**Table 2:** Inclusion and exclusion criteria

Inclusion criteria	Exclusion criteria
1. Over 18 years of age who reside in the province of Corrientes at the time of diagnosis	1. Pregnant or breastfeeding women
2.Confirmed diagnosis of COVID-19 by polymerase chain reaction test for detection of SARS-CoV2 in the last 48 h	2. Known allergy to ivermectin or some of the components of ivermectin tablets or placebo
3. In case of being women of childbearing age, they must be using a contraceptive method of proven efficacy and safety (barrier, hormonal, or permanent contraceptives) for at least 3 months prior to inclusion in the present study, during the entire period of time for the duration of the study, and until at least 30 days after the end of this study. A woman will be considered to have no reproductive capacity if she is postmenopausal (at least 2 years without her menstrual cycles) or if she has undergone surgical sterilization (at least 1 month before the time of inviting her to participate in this study)	3. Current use of home oxygen
4. Weight at the time of inclusion equal to or greater than 48 kg	4. That require hospitalization due to COVID-19 at the time of diagnosis or history of hospitalization for COVID-19
5. That they sign the informed consent for participation in the study	5. Presence of mal-absorptive syndrome
	6. Presence of any other concomitant acute infectious disease
	7. Known history of severe liver disease, for example liver cirrhosis
	8. Need or use of antiviral drugs at the time of admission for another viral pathology other than COVID-19
	9. Need or use of hydroxychloroquine or chloroquine
	10. Use of ivermectin up to 7 days prior to randomization
	11. Patients on dialysis or who have required it in the last 2 months or who plan to do it in the next 2 months
	12. Current participation or in the last 30 days in a research study that has included the administration of a drug

**Main outcomes:**

Primary outcome will be the percentage of hospitalizations in patients with COVID-19 in the intervention and control groups.

Secondary outcomes: time to hospitalization in each of the arms of the study: number of days elapsed from the inclusion in the study until the hospitalization of the patient; percentage of use of invasive mechanical ventilation in each of the study arms: every patient who is connected to invasive mechanical ventilation after signing the informed consent and before the final study visit; time to invasive mechanical ventilation in each of the arms of the study: number of days elapsed from inclusion in the study to connection to invasive mechanical ventilation of the patient; percentage of patients requiring dialysis in each of the study arms: all patients who require renal replacement therapy of any kind, temporary or permanent, and which begins after signing the informed consent and before the final visit; mortality from all causes in each of the two trial groups: death of the patient, from any cause. Negative PCR swab at 3 ± 1 and 12 ± 2 days after entering the study.

Ivermectin safety: it will be analyzed according to the incidence of adverse events that patients present in the intervention and control groups.

The end of study (EOS) is recorded as the day the patient is discharged or death. Discharge will be granted according to the current recommendations of the Ministry of Public Health of the Province of Corrientes. A follow-up visit (EOF) will be made by phone 30 days after the EOS when vital status will be verified.

**Randomization:**

Randomization will be done through a web system with randomly permuted blocks. Randomization will be carried out by one of the investigators who will not participate in the inclusion of patients or in the delivery of medication (Table 3).

**Table 3:** *EOS* end of study, *EOF* end of follow-up

Visit	Basal and randomization, day 0	Day 3 ± 1	Day 12 ± 2		
	V#1	V#2	V#3	EOS	EOF
Informed consent	X	–	–	–	–
Inclusion/exclusion criteria	X	–	–	–	–
Demographic data and medical history	X	–	–	–	–
Concomitant medication	X	–	–	–	–
Vital signs*	X	X	–	–	–
Anthropometric data^^^	X	–	–	–	–
Basal laboratory	X	–	–	–	–
PCR swab	–	X	X	–	–
Assessment of adverse events	–	X	X	X	–
Final objective evaluation	–	X	X	X	X
Randomization	X	–	–	–	–
Adherence to treatment	X	X	–	–	–

*Includes heart rate, temperature, and oxygen saturation by a digital saturometer

^Includes weight and height

**Blinding (masking):**

The participants, investigators, care providers, and outcome assessors will be blinded.

**Numbers to be randomized (sample size):**

We will include a total of 500 patients (250 patients in each group).

**Trial status:**

This is version 1.0, 17 August 2020. The recruitment started on 19 August 2020, and we anticipate the trial will finish recruitment on 31 December 2020.

**Trial registration:**

ClinicalTrials.gov NCT04529525. Registered on 26 August 2020

**Full protocol:**

The full protocol is attached as an additional file, accessible from the *Trials* website (Additional file 1). In the interest of expediting the dissemination of this material, the familiar formatting has been eliminated; this letter serves as a summary of the key elements of the full protocol.

**Supplementary Information:**

The online version contains supplementary material available at 10.1186/s13063-020-04813-1.

## Supplementary Information

**Additional file 1.** The full protocol

## Data Availability

Not applicable.

