# Supplementary material for: Ivermectin to prevent hospitalizations in patients with COVID-19 (IVERCOR-COVID19): a structured summary of a study protocol for a randomized controlled trial
Source: Trials. 2020 Nov 24;21:965. doi: 10.1186/s13063-020-04813-1 (PMC7683865; doi:10.1186/s13063-020-04813-1)
Supplement: Supplementary file 1 — Additional file 1. The full protocol [file 13063_2020_4813_MOESM1_ESM.pdf]

Ivermectin to Prevent Hospitalizations in COVID-19 (IVERCOR-COVID19) Randomized, double-blind, placebo-controlled

Abstract

Introduction: the coronavirus disease (COVID-19) has become a great concern for everyone. Although multiple investigations have been carried out or are in progress to discover an adequate treatment or prevention, so far we do not have any effective drugs for this disease. Ivermectin, a worldwide antiparasitic, could be effective in this group of patients.

Methods: it will be a single-center, randomized, double-blind study, compared with placebo, to be carried out in the province of Corrientes (Argentina). This study will try to demonstrate the efficacy of ivermectin administered early in patients with coronavirus. This work will have as its primary endpoint the percentage of hospitalizations in patients with coronavirus compared in both arms (ivermectin versus placebo). The secondary points were designed to explore other complications of COVID-19, to evaluate if there is any relationship between the time of administration of the medication and the time in which these complications appear and the early negativization of the swabs.

Conclusions: with this trial we hope to demonstrate that ivermectin can be effective in treating patients with coronavirus administered early to prevent hospitalizations in these patients.

Introduction

The severe acute respiratory syndrome coronavirus disease type 2 (SARS-CoV-2) is a life-changing entity around the world and even more so since the declaration of a coronavirus disease (COVID-19) pandemic on 11 March 2020 by the World Health Organization.

According to official data as of August 30, 2020; COVID-19 has affected more than 25,000,000 people and has been linked to the death of more than 840,000 patients around the world[1]. In Argentina, more than 400,000 cases and close to 8,000 deaths have been reported[2].

It is a pathology that in approximately 80-85% of cases is mild or asymptomatic and around 15% of patients will have more severe forms. The latter are life-threatening and this makes it necessary to have a specific treatment[3].

Since the discovery of this new condition, more than 3,000 officially registered studies around the world have been carried out or are in progress with the aim of finding a

treatment that reduces morbidity and mortality in these patients[4]. So far, numerous antiviral drugs have been studied without achieving conclusive results that allow their widespread use for this pathology[5].

Among the therapeutic options is ivermectin, which has been approved as an antiparasitic[6] but has also been shown to have antiviral activity[7-8-9-10]. This drug has the property of acting by destabilizing the union between the alpha and beta1 subunits of importin. This common intracytoplasmic protein in humans is responsible for transporting different protein messengers to the cell nucleus to trigger a response according to the message received[11-12]. SARS-Cov2 has a protein not yet typed that acts as that messenger and binds to the importin complex to be taken to the nucleus of the host cell where it is intended to trigger an inhibitory response of the cellular mechanism for host defense[13-14]. There is in vitro evidence that destabilization of the junction between the two importin subunits by ivermectin decreases the replication of SARS-CoV2 ribonucleic acid by up to 5,000 times in 48 hours[15] (Figure 1).

Within the spectrum of its current indications, ivermectin has proven to be a safe, effective and cheap drug, which is why the World Health Organization included it in the twenty-first list of essential drugs[16]. Among the few serious adverse effects of this drug is neurotoxicity. This effect is due to the fact that ivermectin has an affinity for chlorine-activated γ-aminobutyric acid (GABA) receptors in invertebrates, exerting one of its mechanisms of action through this pathway in parasitosis[17]. These receptors are also found in the central nervous system of mammals[18], but normally the blood-brain barrier prevents the passage of ivermectin. although in inflammatory processes that can affect the central nervous system and increase the permeability of the barrier, ivermectin could cross it, which could potentially be dangerous for the patient[19-20].

For all this that we have detailed, ivermectin has aroused great interest in the study of its efficacy in patients with COVID-19 to the point that at least 37 studies with this drug are currently under development throughout the world without even the moment there are final results reported in none of them[21].

That is why we have considered that ivermectin could be a therapeutic option in patients with COVID-19 and we have designed this single-center study to be carried out in the province of Corrientes (Argentina) called Ivermectin to prevent hospitalizations in patients with COVID -19 (IVERCOR-COVID19). In this article we detail the fundamentals and design of this project.

Method

Trial objective

To assess the efficacy of ivermectin in addition to standard treatment compared to standard treatment alone in reducing hospitalizations in the COVID-19 patient population.

Study design

It will be a single-center, prospective, randomized, double-blind, placebo-controlled study that will be carried out by the Ministry of Public Health of the Province of Corrientes (Argentina) in coordination with the Institute of Cardiology of Corrientes. The study was evaluated and authorized by the Bioethics Committee in Health Sciences Research of the Faculty of Medicine of the National University of the Northeast (UNNE) and approved by the health authorities of the Province of Corrientes. Patients who meet all the inclusion criteria and none of the exclusion criteria will be randomized via the web system to receive ivermectin or placebo.

The province of Corrientes provides a favorable setting to develop this single-center study because all patients with COVID-19 are treated by a single group of professionals who make up the Crisis Committee of the province. In addition, there is a single hospital throughout the province for the exclusive admission of COVID-19 patients who require it.

Trial interventions

Intervention group: patients who are randomized to ivermectin will receive the dose according to their weight (Table 1) the day they enter the study and 24 hours after the first dose.

Control group: patients who are randomized to placebo will receive the dose according to their weight (Table 1) on the day they enter the study and 24 hours after the first dose.

Both the manufacture of the ivermectin tablets and the placebo will be carried out in the province of Corrientes at the Corrientes Drug Plant.

The doses used in this study are those approved in Argentina for the treatment of parasitosis that require ivermectin.

The tablets should be taken with water or alone; they should not be taken with juices, sodas or any other drink; they are not recommended to be taken with meals or up to 2 hours before or after meals as they can alter the absorption of ivermectin. In the event that the patient refers current gastritis or some degree of digestive intolerance, the tablets may be taken individually with up to 3 hours of difference in intake, but this should only be exceptional depending on the actual intolerance of the patient.

Patient selection

Patients who meet the following criteria will be invited to participate:

Inclusion criteria: These criteria are based on using ivermectin tablets in the population in which it is approved for use in other indications and with the purpose of starting administration as early as possible due to its potential mechanism of action (Table 2) . If we take into account that ivermectin would act on the host cell to improve the

response to virus attack, it is essential to administer it as early as possible while the virus is circulating and not once the inflammatory cascade begins.

Exclusion criteria: These criteria are intended to exclude patients with contraindications to the administration of ivermectin, or who have diseases that may hinder the absorption and bioavailability of the drug. Furthermore, patients who may bias the results of the end points are excluded (Table 2).

Trial outcomes

Primary outcome: it will be the percentage of hospitalizations in patients with COVID-19 in each of the branches of the study. This binary variable was chosen because the hypothesis is that ivermectin acting in the early stage of the disease could decrease the viral load and improve the host's immune response, thus improving the patient's evolution.

Secondary outcomes: These items were intended to explore other complications of COVID-19, premature negativization of nasal swabs, and drug safety. In addition, to assess whether there is any relationship between the time of administration of the medication and the time in which these complications appear because, if the complications in the group with active treatment appear later than in the control group, a new hypothesis it would be that perhaps ivermectin would have to be administered even earlier.

These secondary outcomes include

Time to hospitalization in each of the arms of the study: number of days elapsed from the inclusion in the study until the hospitalization of the patient.

Percentage of use of invasive mechanical ventilation in each of the study arms: every patient who is connected to invasive mechanical ventilation after signing the informed consent and before the final study visit. The need for invasive mechanical ventilation will be defined by the treating physician based on the protocol of the Crisis Committee of the Province of Corrientes and on the recommendations of the Ministry of Health of the Argentine Nation[22].

Time to invasive mechanical ventilation in each of the arms of the study: number of days elapsed from inclusion in the study to connection to invasive mechanical ventilation of the patient.

Percentage of patients requiring dialysis in each of the study arms: all patients who require renal replacement therapy of any kind, temporary or permanent, and which begins after signing the informed consent and before the final visit.

Mortality from all causes in each of the branches of the study: death of the patient, from any cause.

Negative of the swab at 3 ± 1 days and 12 ± 2 days after entering the study: it will be considered when the nasopharyngeal swab is negative on any of these days.

Ivermectin safety: it will be analyzed according to the incidence of adverse events that patients may present in each of the branches.

Recruitment and patient consent

Once the study has started, all patients who meet the inclusion criteria and none for exclusion will be invited to participate in the protocol. This trial has an informed consent that has been approved by the Bioethics Committee in Health Sciences Research of the UNNE School of Medicine, complying with the requirements of national and provincial regulations.

Methods of data collection and the duration of follow-up

Patients with COVID-19 will be contacted by phone to invite them to participate in the study. In case of being interested, one of the researchers will go to the patient's home where they will deliver the informed consent which the patient will eventually sign if they agree to participate together with one of the researchers. The informed consent that is signed by the patient and the researcher will be kept in a sheet to which it will be sprayed with 70% alcohol after the patient delivers it, it will be left in ultraviolet light for 72 hours and then it will be archived.

In the initial inclusion and randomization visit (Visit # 1), patient data related to their demographic characteristics, pathological history, concomitant drug treatment prior to study inclusion, vital signs (heart rate, temperature, oxygen saturation) will be recorded. using digital portable saturation meter, current weight). During this visit, the signs and / or symptoms related to COVID-19 and their start date will be collected; or if the patient is asymptomatic. Among the signs and / or symptoms, it will be considered whether or not the patient had any of the following parameters: body temperature equal to or greater than 37.5 ° C, sore throat, cough, dyspnea, anosmia, dysgeusia or others that at the discretion the investigator may be clinically relevant.

A baseline blood laboratory will be carried out, which will consist of a complete blood count, hepatogram, urea and creatinine, among others. In addition, the patient will be invited to give her consent to store a sample of her blood for possible future studies. This consent has also been approved by the Bioethics Committee in Health Sciences Research of the UNNE School of Medicine.

The medication will be given to you according to the randomization number to which it has been randomized and the dose will be explained according to your weight and how to take it.

The data related to vital signs and saturation will be collected by the researcher who assists the patient, while the rest of the data will be collected by another researcher who will contact the patient by phone.

At the time of performing the second swab at 3 ± 1 days (Visit # 2) after being included in the protocol, vital signs will be recorded again (the same as in Visit # 1). These data will be collected by the researcher who assists the patient. Also at this visit, the researcher who assists the patient will verify adherence to treatment according to the prescription that has been made to the patient and, if there is a remnant of tablets, will ask the patient to deliver them so that the investigator can proceed to its elimination. In addition, all events related to the study end points and potential adverse events will be documented. The patient will also be contacted by telephone to record the evolution or appearance of symptoms related to COVID-19.

12 ± 2 days (Visit # 3) after the patient entered the study, a new swab will be performed, which will be performed by an investigator at the place where the patient is. In addition, another researcher will contact the patient by telephone to collect data related to the objectives of the study and potential adverse events.

It will be recorded as the end of study (EOS) at the moment in which the patient is considered to be discharged, that is, after at least 10 days have elapsed since the symptoms started, with at least 72 hours of disappearance or without worsening of the symptoms. respiratory symptoms, dysgeusia and / or anosmia and 2 consecutive swabs separated by at least 24 hours negative for SARS-CoV2; This definition may be modified according to the epidemiological moment and according to the recommendations of the Ministry of Health of the Argentine Nation. It will be documented if the patient presented any of the final objectives of the study and if he presented any adverse events.

A follow-up visit (EOF) will be made by phone 30 days after the EOS where vital status will be verified.

In each of the face-to-face visits in which an investigator assists the patient, the investigator will have level 3 personal protection equipment.

Table 3 shows the flow chart for each of the visits.

The appearance or worsening of an undesirable sign, symptom or medical condition that occurs in a patient after signing the informed consent will be considered an adverse event, even if it is not related to the study drug or procedure. The de novo presence of any of these that was not present prior to the signing of the consent will be considered as the appearance of an undesirable sign, symptom or medical condition; and as a worsening of the exacerbation of any undesirable medical sign, symptom or clinical condition that makes the patient require a treatment (pharmacological or non-pharmacological) that he did not previously need when signing the consent.

Anyone who causes death or endangers the life of the patient, or that causes or prolongs hospitalization or that can cause persistent or significant disability or disability, or that causes birth defects or birth defects or major medical conditions. In the event that a serious adverse event is the same as a study objective (for example,

hospitalization or death among others), the event will be considered as fulfillment of the final objective and not as a serious adverse event.

The occurrence of SAEs and / or end points will be especially considered in the interim analyzes and reported by the researchers to the Ethics Committee, Steering Committee and provincial health authority, to evaluate the safety and / or efficacy implications that they may have.

Randomization: it will be done through a web system with randomly permuted blocks (of 4 patients, 6 patients and 8 patients). Randomization will be carried out by one of the investigators who will not participate in the inclusion of patients or in the delivery of medication

Analytic plan

The collected variables will be analyzed in such a way that the continuous variables will be expressed as means (± standard deviation) or medians (with interquartile range 25-75) according to their distribution. Categorical variables will be expressed as a percentage and their 95% confidence interval. Continuous variables will be compared in both groups using the Student's t test or the Wilcoxon test according to their distribution. The categorical variables will be compared in both groups using the chi square test. For the analysis of the primary endpoint, the chi square test and logistic regression will be used. For the secondary objectives, the chi square test and logistic regression will be used if the secondary objective is categorical; Student's t test or Wilcoxon test if the variable is continuous and according to its distribution; and the log-rank test with its corresponding Kaplan-Meier curve and the Cox test if the secondary objective is a time variable. Longitudinal analysis will be performed for the analysis of paired samples of both vital signs and nasal swabs at 3 ± 1 and 12 ± 2 days. Regarding the safety point, it will be analyzed using the chi square test and logistic regression, in addition an analysis will be carried out according to the severity of adverse events (serious and non-serious, comparing them according to receiving ivermectin or placebo).

Multivariate analysis of the primary end point will be performed. In the multivariate analysis, the variables that in the univariate analysis had a value of $p < 0.25$ will be included. To choose the best multivariate model, the Hosmer-Lemeshow goodness of fit test, comparison of areas under the ROC curve with the DeLong method, multicollinearity, Akaike's information criterion and Bayesian information criterion will be taken into account.

Subgroup analyzes are planned based on whether patients are symptomatic or asymptomatic; according to age (<65 years or ≥65 years), in symptomatic patients according to the duration of symptoms prior to inclusion in the study (<7 days or ≥7 days).

A value of $p < 0.05$ will be considered significant.

Interim internal analyzes of study objectives and serious adverse events will be performed by including 125; 250 and 375 patients in order to assess the eventual need for an early termination of the study. For these intermediate internal analyzes, the Haybittle-Peto rule will be followed, for which a value of $p < 0.001$ will be considered significant[23]. The results of these analyzes will only be disclosed to all authors and researchers if a value of $p < 0.001$ is achieved. The study will be monitored by a Steering Committee and a Safety Committee. According to the considerations of these last two Committees or in the event that some of the intermediate internal analyzes meet the objective of statistical significance, a report will be submitted to the Committee on Bioethics in Research in Health Sciences of the Faculty of Medicine of the UNNE and to the health authority of the Province that authorized the study to decide whether or not to continue the study. In the event that in any of these intermediate analyzes the value of $p < 0.001$ is reached, the inclusion of patients will be suspended until the Bioethics Committee in Health Sciences Research of the UNNE Faculty of Medicine and the health authority of the province evaluate the results and issue them with respect to them. If these evaluating bodies authorize continuing with the study, patients will continue to be included; otherwise the study will be terminated.

Patients will be analyzed according to the group to which they were assigned during randomization regardless of whether they later received ivermectin or placebo (intention-to-treat analysis).

STATA version 16.0 / SE software will be used for the analysis.

Sample size: It is very difficult to predict the size of the sample since, to date, there are no in vivo studies that have reported data with ivermectin and the proportion of patients requiring hospitalization is very diverse. Assuming that 10% of patients are hospitalized; estimating a level of statistical significance of 0.05, with a statistical power of 80% and calculating a reduction in the odds ratio in the ivermectin branch of between 0.3 and 0.5, between 478 and 1,068 patients in total (50% of the patients in each branch). For this work we will include a total of 500 patients (250 patients in each group).

Study management and governance

This study will have a Steering Committee and a Security Committee for data monitoring, made up of professionals of recognized competence and independent of this work. The Steering Committee will be in charge of monitoring the events, whether they are end points or adverse events, and in the event of a change in their frequency, it will request the Safety Committee to perform the corresponding analysis of the data obtained so far in order to determine if it some relationship to the study drug. If so, the authorities of the Ministry of Health of the Province of Corrientes (Argentina) and the Bioethics Committee in Health Research of the Faculty of Medicine of the UNNE will be informed.

Estimated duration of the trial

It is estimated that it will take 4 to 5 months to include the number of patients needed for the study. This estimate is made based on the increasing rate of COVID-19 cases that the province of Corrientes has had in the last 4 weeks prior to the publication of this trail (25 cases per week).

Dissemination strategy

The results of this work will be immediately reported to the health authorities of the province of Corrientes and the Argentine Nation. In addition, they will be communicated to the entire medical community by publishing the study in medical journals; medical scientific activities of any kind (congresses, seminars, workshops, among others); social networks and community outreach programs.

Limitations

Among the main limitations of the study we find that, up to now, all researchers in the world have learned very little about the behavior of SARS-CoV2; nor do we have data on the use of ivermectin in these patients. But in turn, these two major limitations transform this study into a great opportunity for advancing science, understanding and improving the morbidity and mortality of patients affected by COVID-19.

Furthermore, due to the foregoing, an error may be incurred in estimating the size of the required sample.

There are publications that show that the dose of ivermectin used in vitro to reduce viral replication is up to 35 times higher than the concentration achieved with current doses of ivermectin in humans[24-25]. Although this could be true in vivo, there is no way to corroborate it without a study that targets parameters in the clinical improvement of patients

At the time of publishing this design, our province has a proportion of patients with positive COVID-19 of 250 cases per million inhabitants, which could make it difficult to recruit patients, but taking into account the marked increase in positive cases in the last period of time, the number of cases is expected to increase.

Inclusion of a cohort of young patients results in a larger sample size and this may represent unnecessary treatment in that group of patients, however it will also improve the external validity of the study.

Anticipated results

With this study we hope to show that the early use of ivermectin in patients with a positive swab for SARS-CoV2 significantly reduces the need for hospitalizations, thereby improving their prognosis.

Discussion

IVERCOR-COVID19 is a randomized, double-blind, placebo-controlled study that has been designed in the context of the COVID-19 pandemic with the hypothesis that ivermectin could reduce hospitalizations in these patients.

This study will be carried out entirely in the province of Corrientes (Argentina) because we consider that it presents a favorable scenario for the development of this study. This is based on the fact that all patients with COVID-19 are cared for by a single group of professionals (doctors, nurses, biochemists, kinesiologists) formed for this purpose (Crisis Committee of the Province of Corrientes); that the province has a unique and exclusive hospital for the care of patients with COVID-19 who require hospitalization; and that the province has its own drug manufacturing plant.

All these points will facilitate the inclusion, control and monitoring of patients; obtaining and dispensing medication; and control by the competent health authority.

Ivermectin has demonstrated in vitro biological plausibility for the treatment of COVID-1915, having the advantage, among other things, of being a relatively innocuous, cheap, easy to obtain, simple and short dosage medicine; and that it could potentially be very accessible to everyone at an early stage if it proves effective in treating COVID-19.

Reference

1 https://coronavirus.jhu.edu/map.html

2 https://www.argentina.gob.ar/salud/coronavirus-COVID-19/sala-situacion

3 Hoffmann C. Treatment. In: Kamps BS, Hoffmann C, eds. Covid Reference, Edition 2020-2. SteinHauser Verlag; Amedeo, Germany; 2020. Website www.covidreference.com [accessed 12 April 2020].

4 https://clinicaltrials.gov/ct2/results?cond=COVID-19

5 ŞİMŞEK YAVUZ S and ÜNAL S. Antiviral treatment of COVID-19. Turk J Med Sci 2020;50: 611-9

6 González Canga A, Sahagún Prieto AM, Diez Liébana MJ, et al. The Pharmacokinetics and Interactions of Ivermectin in Humans—A Mini-review. AAPS J. 2008 Mar; 10(1): 42–6

7 Gotz, V., Magar L, Dornfeld D et al. Influenza A viruses escape from MxA restriction at the expense of efficient nuclear vRNP import. Sci. Rep 2016:. 6, 23138.

8 Lundberg, L., Pinkham C, Baer A, et al. Nuclear import and export inhibitors alter capsid protein distribution in mammalian cells and reduce Venezuelan Equine Encephalitis Virus replication. Antivir. Res. 2013; 100 (3), 662–72.

9 Tay, M.Y., Fraser JE, Chan WKK et al. Nuclear localization of dengue virus (DENV) 1-4 non-structural protein 5; protection against all 4 DENV serotypes by the inhibitor Ivermectin. Antivir. Res. 2013;99 (3), 301–6.

10 Wagstaff, K.M., Sivakumaran H, Heaton SM et al. Ivermectin is a specific inhibitor of importin alpha/betamediated nuclear import able to inhibit replication of HIV-1 and dengue virus. Biochem. J. 2012;443 (3), 851–6.

11 N.C. Chi, E.J. Adam, S.A. Adam, Sequence and characterization of cytoplasmic nuclear protein import factor p97, J. Cell Biol. 130 (1995) 265 – 74

12 D. Gorlich, I.W. Mattaj, Nucleocytoplasmic transport, Science 271 (1996) 1513 – 8

13 Rowland, R.R., Chauhan V, Fang Y, et al. Intracellular localization of the severe acute respiratory syndrome coronavirus nucleocapsid protein: absence of nucleolar accumulation during infection and after expression as a recombinant protein in vero cells. J. Virol 2005; 79, 11507–12.

14 Timani, K.A., Liao Q, Ye L et al.. Nuclear/nucleolar localization properties of C-terminal nucleocapsid protein of SARS coronavirus. Virus Res. 2005;114, 23–34.

15 Caly L, Druce JD, Catton MG, et al. The FDA-approved Drug Ivermectin Inhibits the Replication of SARS-CoV-2 in Vitro. Antiviral Res 2020;178:104787.

16 World Health Organization (2019) World Health Organization model list of essential medicines: 21st list 2019. World Health Organization, Geneva

17 Rang HP, Dale MM, Ritter JM, et al, editors. Rang and Dale's Pharmacology. 6th ed. China: Churchill Livingstone; 2007. p. 715–6.

18 Menez C, Sutra JF, Prichard R, et al. Relative neurotoxicity of ivermectin and moxidectin in Mdr1ab (-/-) mice and effects on mammalian GABA(A) channel activity. PLoS Negl Trop Dis 2012; 6: e1883

19 Varatharaj A, Galea I. The blood-brain barrier in systemic inflammation. Brain Behav Immun 2017; 60: 1–12

20 Poyiadji N, Shahin G, Noujaim D, et al. COVID-19–associated acute hemorrhagic necrotizing encephalopathy: CT and MRI features. Radiology 2020 Mar 31;201187

21 https://clinicaltrials.gov/ct2/results?cond=COVID-19&intr=%22Ivermectin%22

22 https://www.argentina.gob.ar/salud/coronavirus-COVID-19

23 Schulz KF, Grimes DA. Multiplicity in randomised trials II: subgroup and interim analyses. Lancet. 2005;365:1657-1661.

24 Chaccour C, Hammann F, Ramón-García S, et al. Ivermectin and COVID-19: Keeping Rigor in Times of Urgency. Am. J. Trop. Med. Hyg., 2020;1021156–1157

25 Schmith VD, Zhou JJ, Lohmer LR, et al. The Approved Dose of Ivermectin Alone is not the Ideal Dose for the Treatment of COVID-19. Clin Pharmacol Ther 2020;10.1002/cpt.1889. doi: 10.1002/cpt.1889.
